# Antibiotic Treatment Response in Chronic Lyme Disease: Why Do Some Patients Improve While Others Do Not?

**DOI:** 10.3390/healthcare8040383

**Published:** 2020-10-03

**Authors:** Lorraine Johnson, Mira Shapiro, Raphael B. Stricker, Joshua Vendrow, Jamie Haddock, Deanna Needell

**Affiliations:** 1 Principal Investigator, MyLymeData, San Ramon, CA 94583, USA; lbjohnson@lymedisease.org; 2 Analytic Designers LLC, Bethesda, MD 20817, USA; mira.shapiro@analyticdesigners.com; 3 Union Square Medical Associates, San Francisco, CA 94108, USA; 4 Department of Mathematics, University of California, Los Angeles, CA 90095, USA; jvendrow@math.ucla.edu (J.V.); jhaddock@math.ucla.edu (J.H.); deanna@math.ucla.edu (D.N.)

**Keywords:** lyme disease, MyLymeData, borrelia burgdorferi, tickborne disease, machine learning, Global Rating of Change Scale, Likert scale, precision medicine, big data

## Abstract

There is considerable uncertainty regarding treatment of Lyme disease patients who do not respond fully to initial short-term antibiotic therapy. Choosing the best treatment approach and duration remains challenging because treatment response among these patients varies: some patients improve with treatment while others do not. A previous study examined treatment response variation in a sample of over 3500 patients enrolled in the MyLymeData patient registry developed by LymeDisease.org (San Ramon, CA, USA). That study used a validated Global Rating of Change (GROC) scale to identify three treatment response subgroups among Lyme disease patients who remained ill: nonresponders, low responders, and high responders. The present study first characterizes the health status, symptom severity, and percentage of treatment response across these three patient subgroups together with a fourth subgroup, patients who identify as well. We then employed machine learning techniques across these subgroups to determine features most closely associated with improved patient outcomes, and we used traditional statistical techniques to examine how these features relate to treatment response of the four groups. High treatment response was most closely associated with (1) the use of antibiotics or a combination of antibiotics and alternative treatments, (2) longer duration of treatment, and (3) oversight by a clinician whose practice focused on the treatment of tick-borne diseases.

## 1. Introduction

Lyme disease, which is caused by the spirochete *Borrelia burgdorferi* and transmitted via tick bite, is the most common vector-borne disease in the United States. The Centers for Disease Control and Prevention (CDC) estimate that 300,000 new cases of Lyme disease occur annually [1]. Most patients who are diagnosed and treated early are restored to health. However, many patients are not diagnosed early and treatment failures ranging from 10 to 35% have been reported even among those treated promptly [2,3,4,5,6,7,8,9]. 

Different definitions have been proposed to identify this population of patients [10]. Some definitions, such as Post Treatment Lyme Disease Syndrome (PTLDS), were proposed by researchers and are more appropriate for use in the research context, which requires narrow, exclusionary entry criteria suitable for randomized controlled trials [10]. Recently, a more clinically relevant definition has been adopted by the International Lyme and Associated Diseases Society (ILADS), whose clinicians treat most patients with persistent or chronic Lyme disease [11]. A 2015 CDC patient study used the term “chronic Lyme disease” [12]. Our study includes patients with Lyme disease diagnosed by a clinician who remain ill six or more months following antibiotic treatment. These patients satisfy the Shor et. al. definition of previously treated chronic Lyme disease. In this paper, we refer to this group of patients as having chronic Lyme disease (CLD).

Due to a stagnant research environment created by a lack of funding, very little progress has been made to characterize CLD or determine the optimal treatment approach. Critical tasks in Lyme disease research that remain largely unaddressed include characterizing the CLD population, defining the criteria for treatment response, and delineating the factors that negatively or positively affect treatment outcomes. Assessment of treatment efficacy requires prior definition of end points, both for response and nonresponse. Outcomes may include nonresponse, partial response, remission, relapse or recurrence, and recovery.

The first study using data from the MyLymeData registry demonstrated that Lyme disease patients are heterogeneous in their response to antibiotic treatment. This study, which drew on a sample of over 3500 patients, revealed substantial treatment variation among patients and identified three groups of treatment responders using the Global Rating of Change (GROC) scale: nonresponders, low responders, and high responders [13]. Approximately half (53%) of the patients in the study reported some improvement, and more than a third (35%) were “high responders” to antibiotic treatment, underscoring the value of large samples, subgroup analysis, and real-world evidence as standard components of Lyme disease treatment studies. This spectrum of improvement is consistent with reports for most pharmaceuticals due to patient treatment response variation [14].

The present study builds on the previously published MyLymeData GROC research and uses both machine learning and traditional statistical analysis to identify and analyze features that might indicate whether a patient would be a high treatment responder, a low responder, or a nonresponder [13]. An important distinction between the two studies is that while the previous study included all patients in the registry, this study focuses specifically on patients with CLD. We also include another important subgroup—CLD patients who now identify themselves as well. Inclusion of these patients allows us to compare well patients with those who remain unwell based on different demographic, diagnostic, and treatment response criteria.

## 2. Materials and Methods

### 2.1. Data Sources

The MyLymeData patient registry was launched in 2015 and has enrolled over 13,000 patients. It includes an extensive survey response database that has amassed over three million data points. Survey items were drawn from recommended registry data elements of the Agency for Healthcare Research and Quality (AHRQ), prior surveys, and peer-reviewed published literature for Lyme disease and other conditions. Additional sources of survey questions included standard government question banks such as the CDC Behavioral Risk Factor Surveillance System, National Health Interview Survey, National Ambulatory Medical Care Survey, and National Center for Health Statistics, as well as the AHRQ Medical Expenditure Panel Survey, as more fully described in the previous GROC study [13]. Items related to the GROC scale were based on published studies using and validating GROC [15,16]. The beta version of the MyLymeData survey was pilot-tested and revised to improve accuracy and ease of use, as recommended by AHRQ.

To promote participation in the survey, various recruitment strategies were utilized including blogs and social media as well as professional conference presentations about the registry. Participation in the registry is voluntary, and all respondent identities remain strictly confidential. When launched, the MyLymeData patient registry research study was approved by the Advarra Institutional Review Board and subsequently approval has been renewed annually. In addition, the analysis of the survey data for this study was exempted from review by the University of California Los Angeles Institutional Review Board because it did not meet the definition of direct human subject research.

### 2.2. Study Participants

The participants in this analysis consisted of US residents who were clinically diagnosed with Lyme disease and who completed the Phase 1 survey and identified their stage of disease as CLD. The sample included both well and unwell patients. For clarity, we use the term combined sample when both the well and unwell CLD respondents are included in an analysis, as distinguished from the unwell sample or the well sample. Those included in the unwell sample must have answered the GROC items accordingly in the previous survey [13].

**Figure 1:**
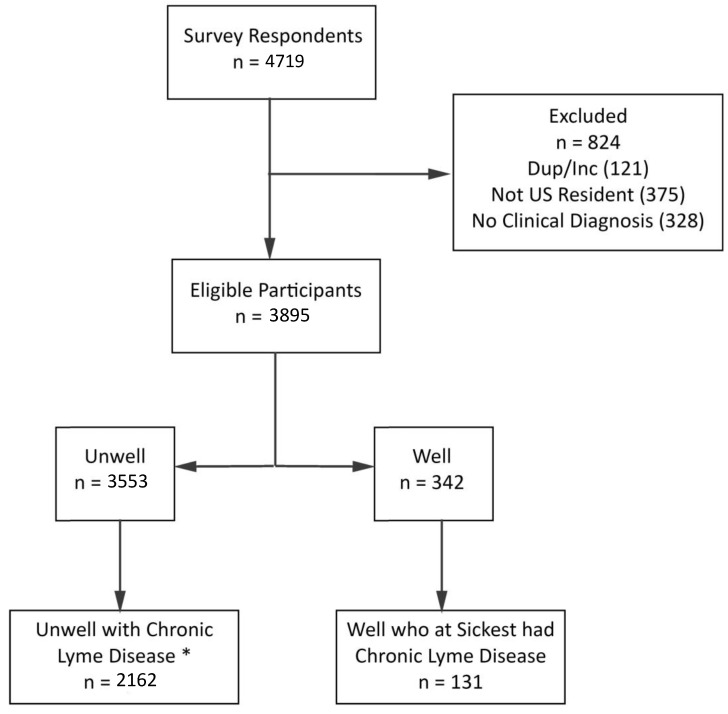
Preliminary sample, exclusions, and final sample size determination. * Chronic Lyme disease participants consist of patients with Lyme Disease who remained ill for at least 6 months following a short-term course of antibiotics. Unwell sample excludes 10 participants who skipped GROC question regarding treatment response.

Figure 1 below shows the original sample of 4719 respondents and the exclusion criteria that resulted in a final sample of 2162 unwell patients and 131 well patients. It is important to bear in mind that patients who are well are less likely to need or seek out a support community or patient registry. Hence, the sample of well patients is likely under-represented here.

#### 2.2.1. Treatment Response Subgroups

The treatment response groups were determined through a series of survey questions. Patients self-identified as being sick or well in a survey question that asked them whether they were sick, well, or somewhere in between (for example, “Ok, but take antibiotics regularly to maintain health or from time to time as needed”). Patients who selected their status as well or in remission were regarded as well; all others were characterized as unwell. Patients who identified as well were given a different branch of the survey from those who were characterized as unwell.

The unwell patients were given a series of GROC scale questions designed to granularly assess treatment response and establish subgroup categories. GROC scales are widely used in research and in clinical practice due to their high face validity, sensitivity to change, and ability to correlate with pain, disability, and quality of life measures [15,16].

The GROC survey questions first asked patients to identify whether their symptoms improved, remained unchanged, or worsened following antibiotic treatment. Patients who responded that they were better or worse were further asked to specify the degree of improvement or deterioration on an eight-point Likert scale. Based on their responses to the GROC questions, each patient was characterized as a high responder, low responder, or nonresponder (see Appendix A).

The sample here included the group of CLD patients comprised of 131 well patients and 2162 unwell patients. The unwell sample was further broken down under GROC into the following subcategories: 947 nonresponders (44%), 396 low responders (18%), and 819 high responders (38%). The 947 nonresponders consisted of CLD patients who, after treatment, reported either being unchanged (634 patients, 29%) or worse (293 patients, 14%). The clinical and diagnostic characteristics of the working sample, within their respective groups, are contained in Table 1 below.

The clinical characteristics between the subgroups did not vary for the most part. However, one substantial difference was the stage of disease at diagnosis, where 29% of the well group reported being diagnosed when they had early Lyme disease compared to 17% of the unwell group. In addition, fewer high responders (26%) reported disability compared to 41% of the nonresponders. Note that all patients were diagnosed with Lyme disease by a clinician and approximately 80% reported a positive lab serology for their diagnosis. The rate of coinfections was similar to that reported in the previous MyLymeData GROC study [13].

Looking across many variables, including those identified in Table 1, there was little difference between nonresponders and low responders. This is because the nonresponder subgroup (44%) consisted of those who reported that, following treatment, they were either worse (14%) or unchanged (29%). Unchanged respondents closely resembled low responders who reported being better after treatment, but only marginally so (“almost the same,” “hardly better at all,” “a little better,” “somewhat better”). In contrast, high responders reported being substantially better (“moderately better,” “a good deal better,” “a great deal better,” and “a very great deal better”). Hence, this sample is best characterized by comparing the answers of nonresponders to those of high responders, and this is the focus of most of our analysis. We include low responders in the Supplementary Data Materials for this study and highlight their data here when distinctions are informative and increase understanding.

To the extent that parallel data exist for patients who are well and unwell, we included well patients either as part of the combined sample or separately to show the spectrum of the disease from those who were the sickest to those who regarded themselves as well. Certain questions, including the GROC questions, were not asked of the well patients. Even where parallel questions were put to the well patient group, the questions may have contained language distinctions to reflect the fact that these patients no longer regarded themselves as ill. For example, a well patient might have been asked, “At my sickest, the stage of my Lyme disease would best be described as…”.

Although this is not a longitudinal study, by including both well and unwell CLD patients here, we have the opportunity to view treatment response across a continuum. At diagnosis, patients may be quite ill or their illness may progress over time. To achieve recovery, these patients will have likely experienced the full spectrum of treatment response, starting as nonresponders and progressing to low responders, high responders, and, ultimately, full recovery. The progress of other patients may stall or stop in any of these categories. Survey responses, for the most part, capture a snapshot of each patient at a point in time on their journey.

#### 2.2.2. Demographics

Table 2 below shows the demographic characteristics of the combined sample.

The sample here was predominantly female (85%) and the average age of all sample participants was 49 years (see Strengths and Limitations below). The sample also skewed more towards higher education and higher income levels than the general population. These demographics conform with other studies of patients with CLD [13,17,18,19].

### 2.3. Methodology Overview

The twin aims of this study were (a) to assess treatment response variation among CLD subgroups based on health indicators and (b) to identify factors that might predict, and ultimately improve, patient treatment response. We first assessed three indicators of health to determine whether they substantially distinguish between these treatment response subgroups: Self-Rated Health Status (SRHS), percentage of symptom improvement, and symptom severity. The aim of this analysis was to determine whether subgroup categories align with expected quality of life measures. For example, did patients who reported being well have a better quality of life, a higher percentage of symptom improvement, and less symptom severity than patients who were unwell?

Quality of life was determined by employing the well-validated and widely used SRHS question which asks patients to rate their general health as excellent, very good, good, fair, or poor. SRHS is part of the CDC Healthy Days measure as well as a survey item in the 36-Item Short Form Survey (SF-36) and is measured annually in the Behavioral Risk Factor Surveillance System (BRFSS) survey [20,21]. Both symptom severity and reported percentage of improvement from baseline illness have been used in varying forms in clinical trials of Lyme disease [17,22,23,24]. These scales are used to measure health quality descriptively as well as to measure treatment outcomes. Hence, they are informative for the comparison of the unwell and well patient subgroups.

We then evaluated the features identified in a companion study using machine learning techniques to determine if survey responses might predict patient treatment response. Readers are directed to this study for further details [25]. The companion study employed multiple machine learning techniques, including basic linear regression, support vector machines, neural networks, entropy-based decision tree models, and K Nearest Neighbors to assess the ability of individual features (responses to survey questions) to predict an unwell participant’s GROC characterization as a nonresponder, low responder, or high responder [25]. The companion study explored 215 features related to diagnostic factors (such as delays in diagnosis, stage of diagnosis, or presence of coinfections), treatment approach, duration of individual antibiotics, alternative treatments, symptoms (severity, presence at time of diagnosis, and three worst), type of clinician, and degree of functional impairment to distinguish between GROC subgroups and to identify the 30 top predictive features (Key Predictive Features). Following this machine learning step, we applied traditional statistical techniques to further characterize how the identified features relate to the patient subgroups. We further analyzed the following variables:

- Treatment approach,
- Treatment duration, and
- Treatment focus of clinician overseeing care.

JMP^®^ 15.0.0 software from SAS^®^ (Cary, NC, USA) was used to conduct all statistical data analyses. The majority of the variables analyzed were categorical and ordinal in nature. To determine the strength, direction, and significance of some of the relationships between variables, Kendall’s Tau-b (alpha = 0.05) was selected. Kendall’s Tau-b is a nonparametric (no assumption of normality required) test that ranks concordant and discordant pairs, resulting in a calculated test statistic that ranges from −1 to 1. This nonparametric statistic was chosen for these analyses since it shows both the magnitude and direction of the relationship and is easy to interpret. A zero value indicates no relationship, a positive value close to 1 represents a strong significant relationship between the two variables that runs in the same direction, and a negative value closest to −1 shows a strong significant relationship between the variables where when one increases, the other decreases. Results are reported with the Tau-b statistic with a 95% confidence interval.

## 3. Results

### 3.1. Indicators of Wellness among Patient Subgroups

In this section, we assess treatment response subgroups across patient-reported health indicators, including SRHS, percentage of symptom improvement, and symptom severity. These questions, which are aimed at determining patient health-related quality of life, are then compared with their treatment response group (including GROC) to determine whether responses reflected variations one might expect within their subgroup.

**Figure 2:**
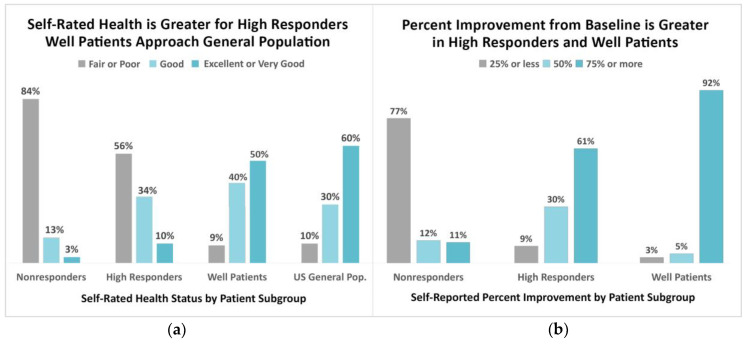
(**a**) Self-rated health is greater in high responders and well patients, with well patients approaching the general population. (**b**) Percentage of improvement from baseline is also greater in high responders and well patients.

Comparison of patient responses to these survey items reveals that patients fall along a continuum dependent upon their treatment response status (well, nonresponder, and high responder). For example, as shown in Figure 2a, on the SRHS, there is a substantial health status increase between nonresponders and those who are well—with the latter approaching the parameters of the general population (τb = 0.40, 95% CI (0.36, 0.44)) [26]

Figure 2b reveals a similar pattern among the treatment response subgroups and the self-rated percentage of improvement since beginning treatment, with the percentage of reported improvement increasing from nonresponders to those who identify as well. These patients were presented with the survey question, “Compared to when I first began treatment, I currently feel…” and given the option to respond along a continuum that ranged from “no better” to more than 85% better. Of those who reported 75% or more improvement, only 11% were nonresponders, compared to 61% of high responders and 92% of well patients (τb = 0.65, 95% CI (0.62, 0.77)).

**Figure 3:**
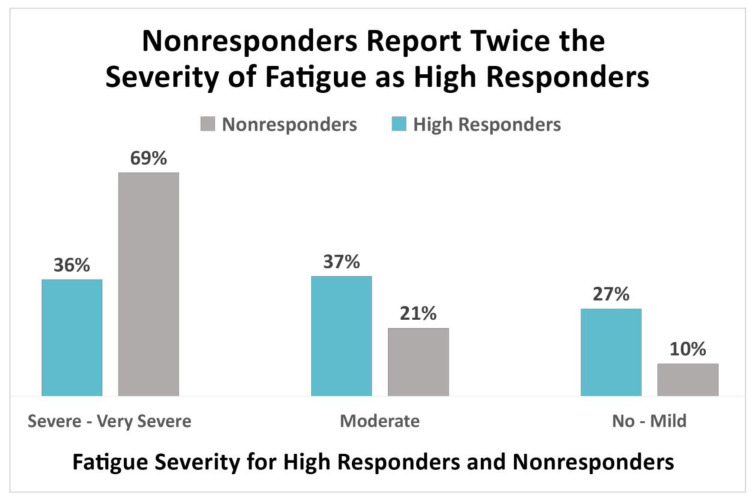
Severe and very severe fatigue is reported by nearly twice as many nonresponders (69%) compared to high responders (36%).

The survey also asked unwell patients to specify the severity of 12 common symptoms of CLD (see Appendix B). Although more extensive symptom lists have been used by others, the list of 12 symptoms used in the registry was created with an eye toward avoiding survey fatigue among participants. As Vendrow et al. report in the companion machine learning study and as Figure 3 demonstrates, the symptom severity for fatigue was substantially less among high responders than among nonresponders [25]. Notably, more nonresponders (69%) reported severe or very severe fatigue compared to high responders (36%) (τ_b_ = −0.33, 95% CI (−0.37, −0.29)).

### 3.2. Predictive Features Associated with Improved Outcomes

Most of the Key Predictive Features identified in the companion machine learning study relate to treatment (20), symptom severity (9), and type of clinician treating Lyme disease. All of the treatment approach questions requested of the unwell patients stem from a general branching question asking whether they were using antibiotics alone, antibiotics and alternative approaches, alternative only, or no treatment. A similar question was asked of the well patients. In this section, we consider factors related to treatment approach or lack of treatment, treatment duration, and clinician type overseeing patient care in the context of the unwell and well sample populations.

#### 3.2.1. The Use of Antibiotics is Associated with High Treatment Response and Wellness

**Figure 4:**
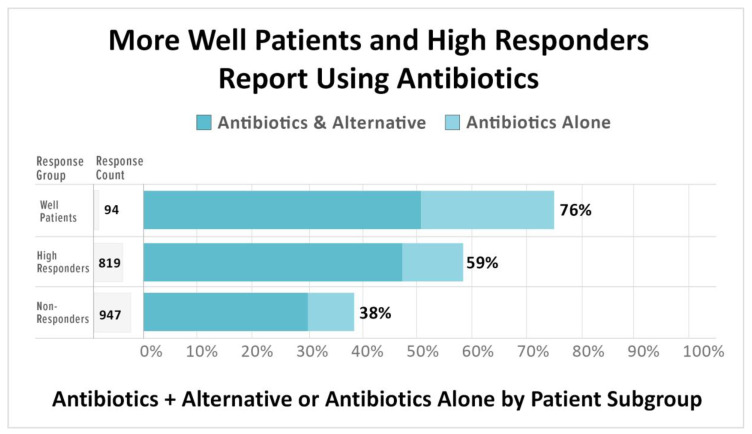
A greater percentage of well patients (76%) and high responders (59%) report using antibiotics or a combination of antibiotics and alternative treatments compared to nonresponders (38%).

Half of the unwell sample reported using antibiotics or a combination of antibiotics and alternative treatments. The remaining 50% of the unwell sample reported using alternative approaches (34%) or no treatment (16%). Antibiotic use is associated with higher treatment response, as Figure 4 indicates.

The majority of well participants (76%) reported that their most effective treatment included antibiotics. Similarly, high responders (59%) also reported being on antibiotics, either alone or in combination with alternative treatments. In contrast, just 38% of nonresponders reported using antibiotics (τ_b_ = 0.23, 95% CI (0.18, 0.27)). Sixty-one percent of nonresponders reported either using only alternative treatments (39%) or no treatment at all (22%). An important question variation should be noted here. Those unwell were asked whether their current treatment approach included antibiotics, while those who identified as well were asked whether the use of antibiotics was among their most effective treatment approaches.

Of those on antibiotics, 84% reported using a single oral antibiotic, with a smaller percentage taking intravenous (5%) or intramuscular (2%) antibiotics. Nine percent reported being on a combination of oral antibiotics and a small percentage reported taking oral antibiotics along with either intravenous or intramuscular. Of those taking antibiotics, 80% percent of the unwell patients and 68% of the well patients reported using a combination of antibiotics and alternative treatments.

Some unwell patients (34%) reported using alternative treatments alone or not using antibiotics or alternative treatments (16%). Many patients not taking antibiotics reported that they were simply on a treatment break (26%) or that they were well or in remission (27%). Another key reason given for not taking antibiotics relates to access to care issues, with 15% reporting no access to treating physicians and 19% reporting insurance company constraints. Others (20%) reported antibiotic treatment side effects as their reason for not taking antibiotics.

A few distinctions among the subgroups bear mentioning. Twenty-five percent of those not taking antibiotics reported that they were never effective—a response reported by far more nonresponders (33%) than high responders (17%). Nonresponders (31%) were also more likely than high responders (17%) to report that they were not using antibiotics because they chose to use other or alternative treatments.

#### 3.2.2. Longer Treatment Durations are Associated with High Treatment Response and Wellness

**Figure 5:**
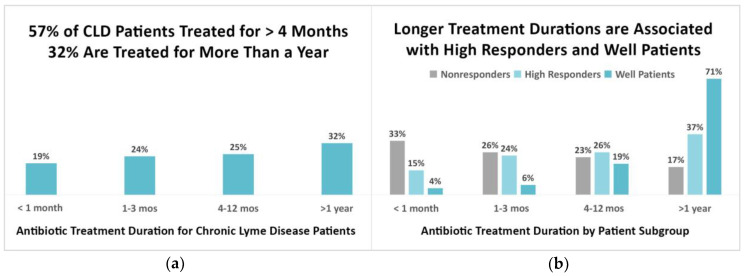
(**a**) Most CLD patients (57%) are treated for more than four months; 32% are treated for more than a year. (**b**) Most high responders (63%) and well patients (90%) report being treated for four months or more, and 37% of high responders and 71% of well patients report being treated for more than a year. Total in (**b**) does not add up to 100% due to rounding errors.

Longer treatment durations were common among the combined sample. As Figure 5a demonstrates, only 19% of those taking antibiotics reported treatment durations of less than one month, with the remainder (81%) reporting longer durations. Most (57%) were treated for four or more months, and 32% were treated for more than a year.

Longer treatment durations were most often associated with high responders and well patients (τ_b_ = 0.33, 95% CI (0.28, 0.36)). Figure 5b shows the treatment durations of nonresponders, high responders, and well patients. Only 4% of those well and 15% of high responders reported treatment durations of less than one month, compared to 33% of nonresponders. Most well patients (90%) and the majority of high responders (63%) reported treatment durations of four or more months, compared to 40% of nonresponders. Seventy-one percent of well patients and 37% of high responders reported treatment durations of one year or more, compared to 17% of nonresponders. Although fewer patients in the combined sample (19%) reported being on treatment regimens beyond two years, a greater percentage of this group (74%) were high responders or well patients.

#### 3.2.3. Clinical Oversight by a Clinician Whose Practice Focuses on Tick-Borne Diseases Is Associated with Higher Treatment Response and Wellness

When respondents in the combined sample were asked to specify the type of clinician overseeing their care, 65% reported that their clinical team included a Lyme-literate medical doctor (LLMD), which the question defined as “a Lyme disease specialist or ILADS-affiliated Lyme specialist.” The remaining 35% selected another type of clinician. Only 11% selected infectious disease specialists.

**Figure 6:**
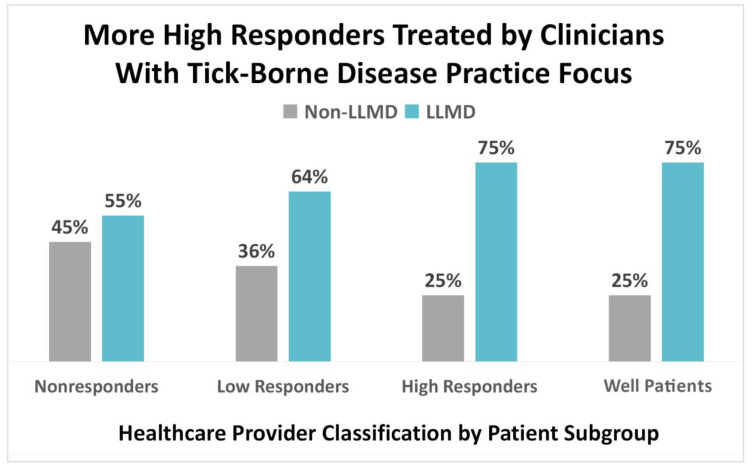
High treatment response is associated with medical oversight by clinicians whose practices focus on tick-borne disease; 75% of high responders and well patients report that their physician was “a Lyme disease specialist or ILADS-affiliated Lyme specialist”.

The type of clinician overseeing patient care varied across categories, although use of an LLMD was associated with greater treatment response, as reflected in Figure 6 below (τb = 0.23, 95% CI (0.19, 0.27)). Most high responders and well patients (75%) reported being treated by an LLMD. Interestingly, the use of LLMDs progressed from nonresponders (55%) to low responders (64%) to high responders (75%). Seventy-five percent of well patients also reported using an LLMD.

## 4. Discussion

### 4.1. The MyLymeData Patient Registry

LymeDisease.org is a grassroots non-profit organization that supports the interests of Lyme disease patients. For over ten years, it has conducted and published peer-reviewed “big data” surveys of patients with Lyme disease [4,5]. In November 2015, it launched the only national Lyme disease patient registry, and since its launch, over 13,000 patients have enrolled. The National Science Foundation awarded a grant to the team of UCLA researchers who are co-authors on this study to explore analytic techniques using registry data. In addition to being a patient registry that collects and analyzes patient data, MyLymeData is a research platform that generates research hypotheses, provides preliminary data to support grants, recruits patients for clinical trials, and facilitates long-term follow-up for these trials after they are completed.

In recent years, patient-centered care and patient-centered research have moved to the forefront of healthcare, and patients now have an emerging voice in the research enterprise [27]. The twin aims of this shift are to improve health outcomes that are most meaningful to patients and clinicians and to meet the demands of stakeholders for real-world evidence [28]. LymeDisease.org developed the MyLymeData patient registry to accelerate the pace of research by building a more robust research capacity utilizing larger samples of patients, less restrictive entry criteria, and real-world data reflecting the wide range of treatment options used in clinical practice.

### 4.2. Patient-Reported Indicators of Health Status

Indicators of wellness and quality of life are broadly used to assess health in general, change in health status, and, when feasible, to compare health status with that of the general population. According to the Food and Drug Administration (FDA), patient-reported outcomes are also widely used in clinical research because “patients are true experts in their disease” [29].

Compared to the general population and patients with other chronic diseases, CLD patients report significantly lower health quality status, more bad mental and physical health days, a significant symptom disease burden, and greater activity limitations [5,23,30,31]. They also report impairment in their ability to work, increased utilization of healthcare services, and greater out-of-pocket medical costs, and, as noted in Table 1, they also report high rates of disability [5,32,33].

The FDA’s Clinical Outcomes Assessment compendium lists approved patient-reported outcomes (PROs) including symptom resolution, improvement and reduction in symptom severity; functional impairment; and health-related quality of life [34]. In a survey of over 6000 Lyme disease patients, more than 95% reported that reduction of symptom severity and improvement in health-related quality of life were either “very important” or “critically important” [35]. Because there are no biomarkers that demonstrate the eradication of *Borrelia burgdorferi* and objective signs are not common in CLD, PROs are the most commonly used outcome measures in clinical trials of this population.

#### 4.2.1. Self-Rated Health Status

The SF-36 is a widely used and highly validated measurement of health-related quality of life. It was included as one of the outcome measures in each of the Lyme treatment trials funded by the National Institutes of Health (NIH) [22,24,30]. A single-item question included in the SF-36, the SRHS, is less burdensome to collect and score than longer scales, which makes it ideal for patient registries. It is also highly validated, reliable, and sensitive to change. Its strong psychometric properties allow it to be used as a substitute for the SF-36 [21].

The SRHS is a particularly useful indicator of health because its broad use in government surveys permits comparisons with the general population and it can be used by researchers, clinicians, and patient registries alike. According to the CDC, SRHS reflects deterioration in health associated with physical functional status and certain chronic illnesses, and it has been shown that lower self-rated health predicts increased mortality [36].

As Figure 2a illustrates, there is a substantial health status increase along the continuum between nonresponders and those who are well on the SRHS. Significantly, well patients report that their health status on the SRHS is comparable in all categories to the general population [26]. The importance of this cannot be over-stated because the fact that well patients compare favorably with the general population contradicts the conclusion by some researchers that antibiotic treatment of these patients is ineffective, regardless of the type or duration of treatment used. Buried within this conclusion is the implicit assumption that these patients are “incurable”. As we discuss below, the majority of well patients were treated with antibiotics for extended periods of time.

In contrast, the percentage of nonresponders who report their health as “fair” or “poor” greatly exceeds the general population (84% versus 10%). High responders fall in between nonresponders and well patients when compared to the general population. As Figure 2a demonstrates, nonresponders report lower health status than high responders, and well patients report even better health status. Although this is not a longitudinal study and cannot be used to demonstrate cause and effect, the association between treatment response and health status is what one would expect as patients progress on the path from being unwell to attaining wellness.

#### 4.2.2. Self-Rated Percentage of Improvement

The results for percentage of improvement, for which responses were measured on a scale ranging from less than 25% improvement to greater than 85% improvement, also align along the expected continuum from sickness to health. This question is similar to one asked by Donta in his CLD clinical studies, which defined “significant improvement” as 75% or greater on his self-rated improvement scale [17,18]. As Figure 2b shows, 75% or more improvement was reported by 92% of well patients and 61% of high responders compared to only 11% of nonresponders. In contrast, very little improvement (25% or less) was reported by most nonresponders (77%). The fact that groups of patients report substantial improvement over baseline since treatment is important for clinicians, researchers, and patients to understand [37].

The minimal clinically important difference for improvement in the CLD population has not been determined [38]. Nonetheless, it is likely that patients who experience a 75% or greater improvement in their condition would consider this amount of improvement to be clinically meaningful. It is encouraging that the response results on this measure generally conform to the pattern of treatment improvement seen in the SRHS findings.

#### 4.2.3. Symptom Severity

The last indicator of wellness that we examined was symptom severity. Symptom severity scales are widely used in many diseases as outcome measures and a number of different symptom scales have been used in persistent Lyme disease trials. A recently published symptom severity questionnaire for “Post Treatment Lyme Disease” was validated against the SRHS and other widely used PROs [23]. Other studies have demonstrated that severity of symptoms is a defining feature of CLD and identified fatigue as the most commonly reported severe symptom [5,10]. Another study identified fatigue as the most important contributor to physical functioning in some Lyme disease patients [39].

Fatigue symptom severity was the second most highly ranked of the Key Predictive Features identified by Vendrow et al. in the companion machine learning study [25]. Almost twice as many nonresponders (69%) reported severe or very severe fatigue compared to high responders (36%). Correspondingly, almost three times as many high responders as nonresponders report that their fatigue either does not exist or is mild. This survey item was not given to the well population on the assumption that symptoms had largely resolved for subjects who identified as well. Analysis of the severity of other symptoms is beyond the scope of this study.

The reduced fatigue severity reported here is consistent with the findings of two NIH-funded Lyme disease retreatment trials that demonstrated efficacy of intravenous ceftriaxone for post-treatment Lyme fatigue [22,24,40]. In the Krupp study, for example, 64% of patients who received antibiotic treatment performed better on the fatigue symptom severity scale used in this study compared to 18.5% of patients who received placebo. Although these studies found antibiotics to be effective in reducing the severity of participant fatigue, neither recommended intravenous treatment in the ordinary course due to concerns about adverse events associated with intravenous antibiotic administration.

What this portion of the study demonstrates is that nonresponders fare much worse on every measure of wellness reviewed, while high responders and well patients perform markedly better—with the well population performing comparably to the general population on the SRHS. As observed in the previous MyLymeData study, GROC is a particularly useful outcome measure because it is simple enough to use in a patient registry and can also be used by clinicians and researchers to assess treatment response [13]. The ability of GROC to differentiate the patient subgroups and yield consistent results with other recognized PROs validates the use of GROC in patients with CLD. It also validates the use of the patient subgroups when assessing treatment response.

### 4.3. Features Associated with Improved Treatment Outcomes

As noted above, the companion machine learning study analyzed 215 features from the MyLymeData patient registry and identified key predictive features [25]. Most of the 30 features identified related to treatment, which is the focus of this section. More specifically, we focus on treatment approach, treatment duration, and clinical focus of the treating clinician.

The use of antibiotics and the duration of antibiotic treatment for CLD is controversial [41]. This is because there is no biomarker that can determine whether *Borrelia burgdorferi* has been eradicated in CLD patients. In addition, CLD is an under-funded disease that has not attracted pharmaceutical interest in treatment improvement. Only three grants have been funded by the NIH to assess treatment response in Lyme patients who remain ill after a short course of antibiotics—the last was funded over 20 years ago. These trials yielded conflicting results, relied on average treatment effects, employed small samples (ranging from 37 to 129 subjects), and excluded over 89% of patients who sought to enroll [22,24,30].

As a result, the state of the science in Lyme disease is limited, uncertain, and unsettled. In these circumstances, the National Academy of Medicine notes that it is not uncommon for divergent treatment guidelines to be developed [41]. Such is the case in Lyme disease. One set of guidelines was developed by ILADS, whose members treat most CLD patients; the other set of guidelines was developed by the Infectious Diseases Society of America (IDSA) [42,43]. While the ILADS guidelines provide for individualized patient care and permit clinicians to exercise their clinical judgement regarding treatment duration, the IDSA guidelines recommend only short-term antibiotic treatment and state that additional treatment for CLD is not effective.

It is against this backdrop that this study was conducted. To our knowledge, it is the largest observational study of patients using real-world data to analyze the response of CLD patients to treatment.

#### 4.3.1. The Use of Antibiotics Is Associated with Improved Treatment Outcomes

Roughly half of the unwell sample reported using antibiotics or a combination of antibiotics and alternative treatments. High responders (58%) and well patients (76%) were more likely to report using antibiotics. In contrast, antibiotic use was far lower in nonresponders (38%). The fact that patients who are high responders or well are more likely to have used antibiotics means that it is not accurate to say that antibiotics are ineffective. It would be more accurate to say that patients vary in their response to treatment and that antibiotic treatment appears to be effective for some subgroups of patients and not others.

The CLD patients who do not take antibiotics provide several reasons. Some factors such as a preference for alternative approaches may reflect patient values. However, access to care issues that interfere with the ability to obtain treatment that appears to be effective for some patients should be eliminated. All patients should have access to potentially beneficial treatment for a disease that carries such a high symptom burden, reduced quality of life, and functional impairment.

A quarter of the unwell sample who were not taking antibiotics reported that antibiotics were never effective for them. More nonresponders (33%) reported this than high responders (17%). Medical treatments are not uniformly effective and nonresponse in certain patients exists in all domains of medicine. Many factors may play a role, including demographic factors, antibiotic choice, the need to use combination treatment approaches, the presence of other undiagnosed tick-borne coinfections, pharmacogenetics (fast or slow drug metabolism), initial severity of disease, stage of disease at diagnosis, natural course of the disease, misdiagnosis of Lyme disease, ability to tolerate side effects, noncompliance, the species of *Borrelia* contracted, the skillfulness of their clinician, and insufficient duration of treatment at the point in time at which they responded to the GROC survey item. It is beyond the scope of this paper to evaluate why a patient might not respond to treatment.

#### 4.3.2. Longer Treatment Durations Associated with Greater Treatment Response

CLD patients using antibiotics report longer treatment durations than those recommended by the CDC and the IDSA [43]. CLD patients who were treated for less than a month were more likely to be nonresponders. The majority of patients (57%) reported treatment durations of four or more months, and 32% reported treatment for more than a year. High responders reported longer treatment durations than those who were nonresponders, and well patients reported even longer treatment durations. Treatment durations exceeding one year were not uncommon among well patients (71%) or high responders (37%). Fewer patients in the combined sample (19%) reported being on treatment regimens beyond two years, but 74% of those who did so were high responders or well patients.

Longer treatment durations were also reported in a CDC HealthStyle national patient survey that found 60% of Lyme disease patients being treated for five or more weeks and 36% for more than 8 weeks [12,44]. Similarly, longer durations were reported in an earlier survey of physician preferences as well as other observational trials that have reported benefits from providing CLD patients with longer treatment durations [17,45,46,47]. Prolonged antibiotic treatment ranging from 6 months to more than 5 years is recommended by infectious disease experts for a number of other conditions [48,49,50,51,52].

When comparing these results to the NIH treatment trials, it is important to bear in mind that none of those trials employed treatment durations exceeding 90 days [22,24,30]. Although the CDC recommends better education for clinicians regarding the appropriate duration of therapy, the results of our study and other observational treatment trials suggest that longer treatment durations may be more effective for certain patients. If antibiotic treatment beyond four weeks was not effective, we would not expect longer treatment durations to be associated with higher response rates as shown in our study.

#### 4.3.3. Clinical Focus of Treating Practitioner

Most respondents in the combined sample (65%) reported that their clinical team included an LLMD, while very few (11%) selected infectious disease specialists. The use of an LLMD was associated with greater treatment response, as reflected by the fact that most high responders and well patients (75%) relied on LLMDs for care.

Some studies have noted a consistent relationship between physician practice volumes and outcomes. This is true for both procedure-based care as well as medically managed care [53]. The practice of an LLMD likely includes a higher volume of patients with CLD, and the improved outcomes associated with LLMDs may reflect the knowledge and expertise gained by treating a larger volume of patients with CLD. This would be consistent with findings by Ziska et al. that longer treatment durations in Lyme disease were prescribed by physicians who had a higher volume of Lyme patients [45]. In this study, treatment durations were also affected by specialty, with longer durations reported by internists in contrast to infectious disease physicians and rheumatologists.

Beyond this, a meta-analysis of 45 studies suggests that trust in the treating clinician may play a pivotal role [54]. In a survey of over 5000 Lyme disease patients conducted by LymeDisease.org in 2011, 77% of respondents reported that they did not trust the treatment guidelines of the IDSA and 73% reported that they select physicians based on whether they follow the guidelines of ILADS [55]. Over 95% stated that it was “essential” or “very important” that guidelines provide patients with treatment options, allow clinicians to use their clinical judgment, provide individualized care, and provide for shared medical decision-making with the patient [4,55]. Each of these elements is included in the ILADS guidelines.

This low level of trust in the IDSA guidelines and strong preference for selecting physicians who follow the ILADS guidelines is consistent with the low percentage of patients who report using infectious disease physicians to treat CLD. The definition of CLD here requires that patients remain ill for at least six months after antibiotic treatment. Hence, the short-term antibiotic treatment recommended by the IDSA has not been effective for these patients. Given the trust issues with IDSA guidelines and failure to respond to short-term antibiotic protocols, patients who select an LLMD to oversee their care may be doing so to obtain access to a greater variety of treatment options than the IDSA guidelines currently provide.

## 5. Strengths and Limitations

The strengths and weaknesses of this study in large measure reflect those inherent in observational trials when compared to randomized controlled trials (RCTs). RCTs are uniquely valued because of their ability to determine cause and effect, but they do so in a process that is inherently time intensive and expensive, typically measuring the effect of a single intervention at a time and generating knowledge sequentially. As one researcher noted: “It can take more than a decade for a trial to progress from the idea stage to actionable information, and cost and complexity mean that many questions will never be addressed with such trials” [56].

In addition, small exclusionary RCT trials, such as the NIH CLD treatment trials, are not generalizable to most patients seen clinically. In contrast, patient registries reflect real-world behavior and practices and employ fewer inclusion and exclusion criteria [57]. Studies with small sample sizes also preclude the type of subgroup analysis essential to identify treatment responders and only are able to detect large treatment effects [40]. Sample sizes in the thousands or tens of thousands are required to detect the small or moderate treatment effects that most patients would regard as meaningful [58,59]. Although a treatment may be clinically significant, the signal may not be apparent in a small study sample [60].

When the optimal treatment, duration, or combination of treatments is unknown, as it is in Lyme disease, the process of conducting back-to-back sequential randomized controlled trials to determine the best treatment approach is not realistic [61]. To accelerate the slow pace of research and to provide real-world evidence, observational data like those collected in patient registries hold enormous appeal for the type of detailed analysis essential to precision medicine [62].

However, registries also pose unique challenges because patients are recruited directly in situations where the underlying sampling frame is unknown [57], and registry samples are often self-selected (as is the case with MyLymeData). These participants have access to the Internet and are not a randomly drawn sample. Those who elect to participate may have been sick longer and more severely ill, which could lead them to seek online support and resources for their illness [5]. Accordingly, we believe that the results reported here capture a segment rather than the full spectrum of patients with Lyme disease. MyLymeData patient registry results are based on self-reported information without independent diagnostic confirmation. However, self-reported information may improve the accuracy of patient data and has been found to have acceptable levels of reliability when compared to medical chart information [57,63,64,65,66].

Participants in this study were predominantly women (85%). Most studies of CLD report a predominance of women who are enrolled, and the percentage of women can range from 66 to 83% [17,18,30,67]. Further studies are needed to determine whether more women develop CLD compared to men and whether avoidable causes such as delayed diagnosis are responsible for this imbalance [68].

Some researchers have raised general concerns regarding the potential for data dredging in “big data” studies using subgroup analysis to assess treatment response heterogeneity [62]. However, this study has followed the recommendations for addressing potential pitfalls by using the limited subgroups defined a priori in a previous study [13]. We also demonstrated that the subgroups performed in the expected and pre-specified directions for the indicators of wellness, and we have interpreted our results cautiously. Although this study includes data from approximately 2300 patients with CLD and has substantial implications for research design and health policy, observational studies such as this are not suitable for determining cause and effect.

## 6. Conclusions

Medical decisions are made for individuals, and assessment of the heterogeneity of treatment effects is critical as medicine seeks to become more personalized and patient-centered [69]. The real-world outcomes presented here demonstrate that patients vary considerably in treatment response and that the use of antibiotics, the duration of treatment, and the type of clinician overseeing care play an important role in determining treatment response. High responders and well patients report substantially higher quality of life, a greater percentage of improvement, and reduction in symptom severity burden. High responder and well patient categories are also associated with antibiotic treatment, longer treatment durations, and treatment by clinicians whose practice focuses on tick-borne diseases. Comparing treatment outcomes between CLD patient subgroups (nonresponder, low responder, high responder, and well patients) supports what is reported by some clinicians, namely that given antibiotic treatment for a sufficient period of time, patients with CLD can improve—and some will improve enough to identify themselves as being well.

Finally, the existence of a heterogeneous patient group suggests that individualization may be more effective than standardization of treatment approach. Until reliable biological markers for the disease are developed, there may be no substitute for the exercise of clinical judgment by physicians who can assess the individual patient’s actual response to treatment to determine the appropriate duration of antibiotic therapy.

## Figures and Tables

**Table 1:** Clinical and diagnostic characteristics of working sample.

Variable	Unwell %	Non Responder	Low Responder	High Responder	Well
Chronic Lyme disease ^a^	100%	100%	100%	100%	100%
**Stage When Diagnosed**					
Late untreated Lyme disease ^b^	77%	76%	78%	78%	66%
Early Lyme disease ^c^	17%	18%	16%	17%	29%
Don’t know/Other	6%	6%	7%	5%	4%
**Key Diagnostic Factors**					
Clinician diagnosed ^d^	100%	100%	100%	100%	100%
Recollection of tick bite	43%	44%	45%	42%	40%
Recollection of EM rash ^e^	35%	34%	37%	36%	30%
With supportive lab tests	80%	81%	77%	79%	79%
1 or more coinfection	70%	62%	69%	78%	63%
**Disabled** (with or without disability benefits)	35%	41%	38%	26%	NA

^a^ Remained ill for 6 months or more after treatment with antibiotics for 10–21 days. ^b^ Remained undiagnosed and untreated for 6 months or more after symptom onset. ^c^ Tick bite, rash, or early Lyme disease. ^d^ To be enrolled, patients must have had self-reported US residency and diagnosis by a healthcare provider. ^e^ Because of a branching error in the initial survey, patients were re-asked this question: these data include the 707 who responded to the revised question. NA, not applicable. EM, erythema migrans.

**Table 2:** Demographic characteristics of respondents.

Variable	Count (% of CLD Well and Unwell)
**Gender ^a^**	
Female	1943 (85%)
Mean age	49
**Education ^b^**	
High school or less	177 (8%)
Some college or associate degree	727 (33%)
Bachelor degree	695 (32%)
Graduate school degree	577 (27%)
**Family income ^c^**	
Less than $25 k	295 (17%)
$25–50 k	308 (17%)
$50–75 k	322 (18%)
$75–100 k	227 (13%)
>$100 k	631 (35%)
**Geography ^d^**	
East	594 (26%)
Midwest	317 (14%)
South	624 (27%)
West	737 (32%)

^a^ 2 selected “other.” ^b^ 117 skipped or selected “prefer not to answer.” ^c^ 510 skipped or selected “prefer not to answer.” ^d^ Excludes 21 who selected either a US territory or who did not indicate a state.

## Supplementary Materials

Data used in the preparation of this article were obtained from the LymeDisease.org patient registry, MyLymeData, Phase 1 27 April 2017. Data and supporting calculations in this report are included as supplementary materials available at https://doi.org/10.6084/m9.figshare.13040636.

## Abbreviations

GROC	Global Rating of Change
LLMD	Lyme-literate medical doctor
CLD	chronic Lyme disease
AHRQ	Agency for Healthcare Research and Quality
BRFSS	Behavioral Risk Factor Surveillance System
SRHS	Self-Rated Health Status
SF-36	36-Item Short Form Survey
AB	Antibiotics
ALT	alternative treatments
PRO	patient-reported outcome
CDC	Centers for Disease Control and Prevention
IDSA	Infectious Diseases Society of America
ILADS	International Lyme & Associated Diseases Society

## Appendix A. The Global Rating of Change Scale

**This question is asked only of those patients who are categorized as unwell.**

As asked, the GROC question produces a 17-point Likert scale. It is a two-part question asking first if the patient is “better”, “worse”, or “unchanged”. Patients who responded that they were better or worse were asked to specify the degree of improvement, ranging from “almost the same” to “a very great deal better/worse”. “Almost the same” responses for better or worse were combined with the unchanged response. As modified, the resultant 15-point Likert scale ranged between −7 and 7, with 0 as the midpoint for unchanged.

We separated participants into three categories:

1. Nonresponders, who answered between −7 and 0, indicating that there was no improvement.
2. Low responders, who answered between 1 and 3, indicating that there was slight improvement.
3. High responders, who answered between 4 and 7, indicating that there was substantial improvement.

In general overall, I would say that with antibiotic therapy, my Lyme symptoms are…

- Worse—→How Much Worse
- Better—→How Much Better
- Unchanged includes “Almost the same” for both Worse and Better responses

**If answer, better:**

More specifically, I would say my degree of health improvement since I began treatment is…

1 Almost the same

2 Hardly better at all

3 A little better

4 Somewhat better

5 Moderately better

6 A good deal better

7 A great deal better

8 A very great deal better

**If answer, worse:**

More specifically, I would say my degree of health decline since I began treatment is…

1 Almost the same

2 Hardly worse at all

3 A little worse

4 Somewhat worse

5 Moderately worse

6 A good deal worse

7 A great deal worse

8 A very great deal worse

## Appendix B. Common Symptoms of Chronic Lyme Disease

- Fatigue
- Headache
- Joint Pain
- Muscle aches
- Neuropathy
- Twitching
- Memory Loss
- Cognitive Impairment
- Sleep Impairment
- Psychiatric
- Heart related
- Gastrointestinal
